# Photodynamic Therapy for Diffuse Choroidal Hemangioma in Sturge-Weber Syndrome

**DOI:** 10.1155/2014/452372

**Published:** 2014-05-07

**Authors:** Sílvia Monteiro, Inês Casal, Marinho Santos, Angelina Meireles

**Affiliations:** Department of Ophthalmology, Centro Hospitalar do Porto, EPE, Largo Professor Abel Salazar, 4099-001 Porto, Portugal

## Abstract

*Purpose.* To report the treatment outcome of photodynamic therapy with verteporfin (PDT) for exudative retinal detachment (RD) associated with diffuse choroidal hemangioma in Sturge-Weber syndrome (SWS). *Methods.* An interventional case report of a 10-year-old girl with SWS who developed an exudative RD (visual acuity hand motions) that was treated with PDT. She was treated with a first session of multispot PDT. Posteriorly, a choroidotomy for drainage of subretinal fluid was created, combined with an intravitreal injection of gas (SF_6_) and cryoapplication. Finally, a second session of PDT was applied. *Results.* Subretinal fluid resolved over a period of one year and visual acuity increased to 20/125. *Conclusions.* PDT is an effective therapeutic option for exudative RD associated with diffuse choroidal hemangioma.

## 1. Introduction

Sturge-Weber syndrome (SWS) is a rare sporadic disorder that occurs with a frequency of approximately 1/50 000 births [1, 2] and is characterized by cutaneous angioma in a trigeminal distribution, leptomeningeal angioma, and choroidal hemangioma [3]. Choroidal hemangioma is an uncommon benign vascular tumor that can be either circumscribed or diffuse [4]. In SWS, choroidal hemangiomas are usually diffuse, unilateral, and ipsilateral to the angiomatous malformation of the skin [5]. Patients with diffuse choroidal hemangiomas are most likely to develop secondary retinal detachment with shifting of the subretinal fluid [5, 6]. Diffuse choroidal hemangioma can lead to visual loss due to refractive errors, foveal distortion, and exudative retinal detachment [7]. Diffuse choroidal hemangiomas have been treated with radiotherapy, proton beam, stereotactic radiotherapy, plaque radiation therapy, and photodynamic therapy (PDT) [5]. PDT is currently being advocated for circumscribed choroidal hemangioma with good short-term results [7]. To date, there have been only seven case reports of successful PDT treatment for diffuse choroidal hemangiomas [4, 5]. In this report we present our experience of treating a choroidal hemangioma with PDT in the setting of SWS.

## 2. Case Report

**Figure 1:**
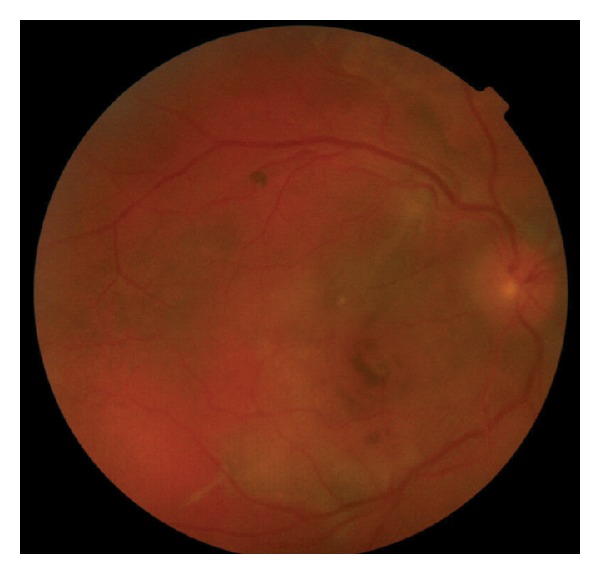
Ophthalmoscopy revealed a tomato ketchup red appearance of the fundus and an exudative retinal detachment.

**Figure 2:**
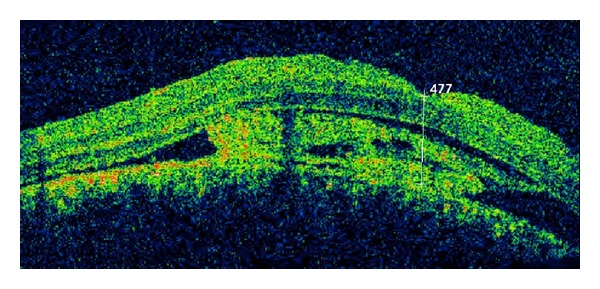
OCT image confirmed the presence of a diffuse choroidal hemangioma with thickening of the choroid and associated RD.

**Figure 3:**
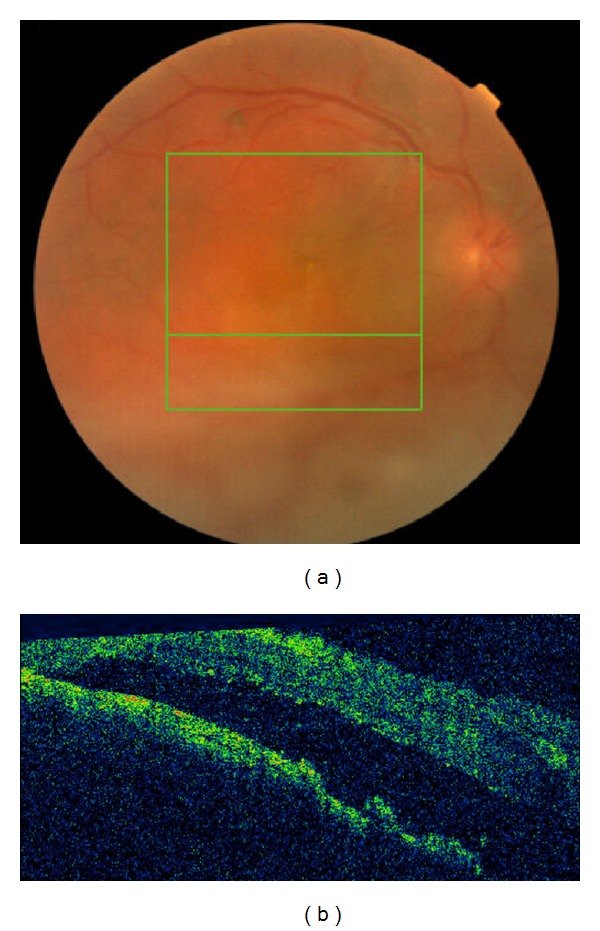
Retinography (a) and OCT image (b) documented the amount of subretinal fluid in the lower retina.

**Figure 4:**
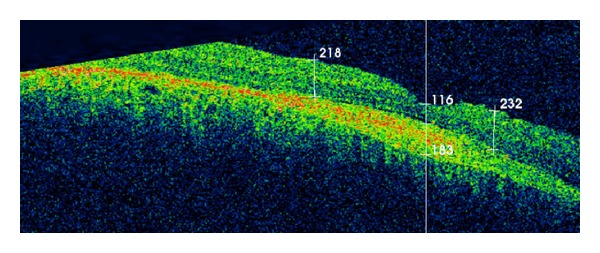
OCT image confirmed the elimination of subretinal fluid and the overlying RD after the second session of PDT.

A 10-year-old white girl with right-sided SWS was diagnosed with right anisometropic amblyopia at age of 6 years and responded to glasses with a stabilized visual acuity of 20/100 in the right eye. She underwent laser treatment of the cutaneous nevus flammeus of the right side of the face with a good cosmetic result. There was no history of glaucoma. Magnetic resonance imaging of the brain excluded intracranial involvement. She presented with decreased vision in the right eye to over a period of 6 months with a hyperopic shift from 4.00 D to 6.00 D. At presentation, her best-corrected visual acuity (BCVA) was hand motions right eye and 20/20 left eye. Intraocular pressures were normal without evidence of iris neovascularization or abnormal conjunctival or scleral vessels. Ophthalmoscopic evaluation revealed a tomato ketchup red appearance of the right fundus compared with the left and a serous retinal detachment (RD) of the right eye (Figure 1). The entire inferior retina of the right eye was detached with involvement of the macula. B-scan ultrasound and optical coherence tomography (OCT) (Figure 2) confirmed the presence of a diffuse choroidal hemangioma with thickening of the choroid and associated RD. Fluorescein angiography showed diffuse early hyperfluorescence. The intense early hyperfluorescence persisted through the laminar venous and full venous phases and only faded in the late angiogram phase. There was no fluorescein leakage from the retinal circulation. The patient underwent PDT with parameters typically used in treatment of choroidal neovascularization. Two sessions of PDT where the tumor was thicker were planned: the macula area and the area next to inferotemporal arcade. Verteporfin was infused at a concentration of 6 mg/m^2^, and an 83-second treatment was conducted with a 689 nm Zeiss laser that was delivered at 50 J/cm^2^ with an intensity of 600 mW/cm^2^. Firstly, four spots of 5300 *μ*m were applied to the diffuse choroidal hemangioma in the macular area. OCT was used to monitor treatment response. Two weeks after treatment, there was a decrease in the thickness of the choroidal tumor. However, because the amount of subretinal fluid (mainly in lower quadrants) has not changed (Figure 3), an inferotemporal choroidotomy for drainage of subretinal fluid was created. This procedure was combined with an intravitreal injection of gas (SF_6_) and cryoapplication in the temporal quadrants. No complications were noted. Forty days later, a second session of PDT treatment was applied. Five spots of 5200 *μ*m were applied out of the inferotemporal arcade. The further PDT session resulted in the total elimination of the subretinal fluid and the overlying RD (Figure 4). One year after the treatment, the RD has not recurred and BVCA improved to 20/125 right eye. No side effects were noted.

## 3. Discussion

Histologic examination of eyes in patients with SWS has suggested an incidence of choroidal hemangiomas of up to 40% [8]. Exudative RD is one of the vision-threatening complications. The management of RD in SWS is often difficult. There are several ways to manage diffuse choroidal hemangiomas. These include various modes of radiotherapy and photodynamic therapy. Radiation and proton beam therapy are effective modalities for diffuse hemangiomas but can only be administered in specialized centers. Potential complications include radiation retinopathy, optic neuropathy, macula ischemia, and subretinal fibrosis [5]. Seven recently published reports indicate that PDT with verteporfin can be used as a therapeutic option for exudative RD associated with diffuse choroidal haemangioma [4, 5]. Results show an effective resolution of subretinal fluid. PDT is a safe and effective modality that has several advantages over other methods. This is an established method of treatment for age-related macular degeneration, and most retina specialists are familiar with its application. As published results of PDT therapy in SWS were favourable and PDT is associated with few systemic or ocular complications compared to the other treatment options, PDT was chosen as a first-line therapy [5].

In this case, after the first PDT session, the amount of subretinal fluid (mainly in the lower quadrants) has not changed. The resolution of subretinal fluid may take up to 6 months after PDT [5, 7]. However, because the amount of retinal fluid was extremely large in the inferior retina, and this area had not yet been treated, the probability of subretinal fluid resolution over time was very low. On the other side, it was not possible to continue the treatment in inferior retina because of poor visualization, consequent difficulty to delimit the area where the tumor was thicker, and define correctly the spots of PDT. In these conditions, PDT would probably be ineffective. For these reasons, an inferotemporal choroidotomy for drainage of subretinal fluid was created to allow the second PDT session planned. Surgery in these eyes is dangerous because of the risk of hemorrhage from the dilated abnormal episcleral and choroidal vessels. To prevent the risk of suprachoroidal hemorrhage, choroidotomy was performed in the inferotemporal quadrant, as lower as possible, at seven o'clock, where the tumor was very little thick. Furthermore, the patient underwent sclerochoroidal diathermy in the same area before external drainage of subretinal fluid. Cryoapplication in the temporal quadrants after the drainage also contributed to preventing the risk of hemorrhage. The risk of retinal incarceration was low because RD was very high. After the transchoroidal drainage of subretinal fluid, the PDT was completed in the inferior retina and allowed the total elimination of the subretinal fluid and the overlying RD.

At this moment no recurring accumulation of subretinal fluid associated with haemangioma in SWS has been reported; however, the follow-up period remains short. Therefore the possibility of retreatment has to be taken into account. We presented one case where PDT resulted in resolution of exudative RD and helped improve visual acuity.

Diffuse choroidal hemangiomas associated with SWS are uncommon but require aggressive treatment to prevent visual loss from exudative RD and possibly subsequent sequelae such as amblyopia. Our experience in this case confirms that PDT with verteporfin is a therapeutic option for exudative RD associated with diffuse choroidal hemangioma. However, long-term follow-up will be necessary to confirm the efficacy of the therapy and to document any further visual recovery.
